# Paired proteomics, transcriptomics and miRNomics in non-small cell lung cancers: known and novel signaling cascades

**DOI:** 10.18632/oncotarget.11723

**Published:** 2016-08-31

**Authors:** Christina Backes, Nicole Ludwig, Petra Leidinger, Hanno Huwer, Stefan Tenzer, Tobias Fehlmann, Andre Franke, Eckart Meese, Hans-Peter Lenhof, Andreas Keller

**Affiliations:** ^1^ Chair for Clinical Bioinformatics, Saarland University, Germany; ^2^ Department of Human Genetics, Saarland University, Germany; ^3^ SHG Clinics, Völklingen, Germany; ^4^ Institute for Immunology, University Medical Center of the Johannes Gutenberg University, Mainz, Germany; ^5^ IKMB, Kiel, Germany; ^6^ Chair for Bioinformatics, Saarland University, Germany

**Keywords:** systems biology, transcriptomics, miRNomics, proteomics, lung cancer

## Abstract

High-throughput omics analyses are applied to elucidate molecular pathogenic mechanisms in cancer. Given restricted cohort sizes and contrasting large feature sets paired multi-omics analysis supports discovery of true positive deregulated signaling cascades. For lung cancer patients we measured from the same tissue biopsies proteomic- (6,183 proteins), transcriptomic- (34,687 genes) and miRNomic data (2,549 miRNAs). To minimize inter-individual variations case and control lung biopsies have been gathered from the same individuals.

Considering single omics entities, 15 of 2,549 miRNAs (0.6%), 752 of 34,687 genes (2.2%) and 141 of 6,183 proteins (2.3%) were significantly deregulated. Multivariate analysis also revealed that effects in miRNA were smaller compared to genes and proteins indicating that expression changes of miRNAs might also have limited impact of pathogenicity. However, a new algorithm for modeling the complex mutual interactions of miRNAs and their target genes facilitated precise prediction of deregulation in cancer genes (92.3% accuracy, p=0.007). Lastly, deregulation of genes in cancer matched deregulation of proteins coded by the genes in 80% of cases.

The resulting interaction network, which is based on quantitative analysis of the abundance of miRNAs, mRNAs and proteins each taken from the same lung cancer tissue and from the same autologous normal lung tissue confirms molecular pathological changes and further contributes to the discovery of altered signaling cascades in lung cancer.

## INTRODUCTION

Integrating and understanding complex data from different high-throughput technologies is a central research topic. Different strategies to uncover genotype–phenotype interactions from multi-omics data sets have been explored as reviewed by Ritchie [1]. Key challenge is the ratio of comparably small cohort sizes (n) and large features sets (p) [2], also referred to as the “small n big p problem” or the “curse of dimensionality”. Integrating different omics data can support discovering true positive events and hence help to reduce the false discovery rate. Factors that contribute to misleading findings are inter-individual variations between cases and controls or the combined analysis of different omics from different cohorts.

To discover and understand altered signaling cascades in lung cancer we chose a study set-up addressing these two challenges. Recently, we already published proteome and transcriptome of 18 cancer and adjacent normal tissue pairs of patients suffering from lung cancer in a proof-of-concept study [3]. To gain deeper insight into the regulative capacity of miRNAs on mRNA and protein levels, we generated miRNA expression in the same samples, improved the proteomic data and performed a novel integrative systems biology analysis of miRNA-, mRNA- and protein profiles. In detail, our results rely on experimental data from 6,183 proteins (compared to 3,328 proteins in the proof-of-concept study), gene expression of 34,687 genes from our previous study and additional 2,549 miRNAs.

We first identified all deregulated features in (all) single omics entities using hypothesis tests (t-test and Wilcoxon-Mann Whitney test). Second, we interactively analyzed all single omics entities using standard methods such as hierarchical clustering or principal component analysis. Third, we mapped all detected deregulated single-omics features, i.e. mRNAs, miRNAs or proteins, onto chromosomes to identify regions enriched for significant features. Fourth, we implemented a novel algorithm to model and determine the mutual influence of miRNAs on their target genes that is based on a simplified stoichiometry. Fifth, we applied the new algorithm to the lung cancer data and showed that the novel approach helps to better understand the regulatory patterns in lung cancer. Finally, we calculated the concordance of gene expression and protein abundance in lung cancer.

## RESULTS

### Dys-regulated miRNAs, genes, and proteins

The discovery of deregulated features is an important goal of case-control omics studies. Common measures for quantifying the degree of deregulation are the t-test and the Wilcoxon-Mann Whitney test. In addition to these hypothesis tests, fold changes or AUC values are frequently calculated. We compared 18 pairs of cancer tissues and unaffected lung tissues from lung cancer patients. Control and cancer tissue biopsies were matched, i.e. for each cancer patient a tumor biopsy and a matched control tissue was available. For the proteomic data (6,183 proteins), transcriptomic data (34,687 genes) and miRNomic data (2,549 miRNAs) deregulation between cancer and control was calculated using the tests and measures mentioned above. The Supplementary Material lists the results for miRNAs (Supplementary Table S1), mRNAs (Supplementary Table S2) and proteins (Supplementary Table S3).

**Figure 1:**
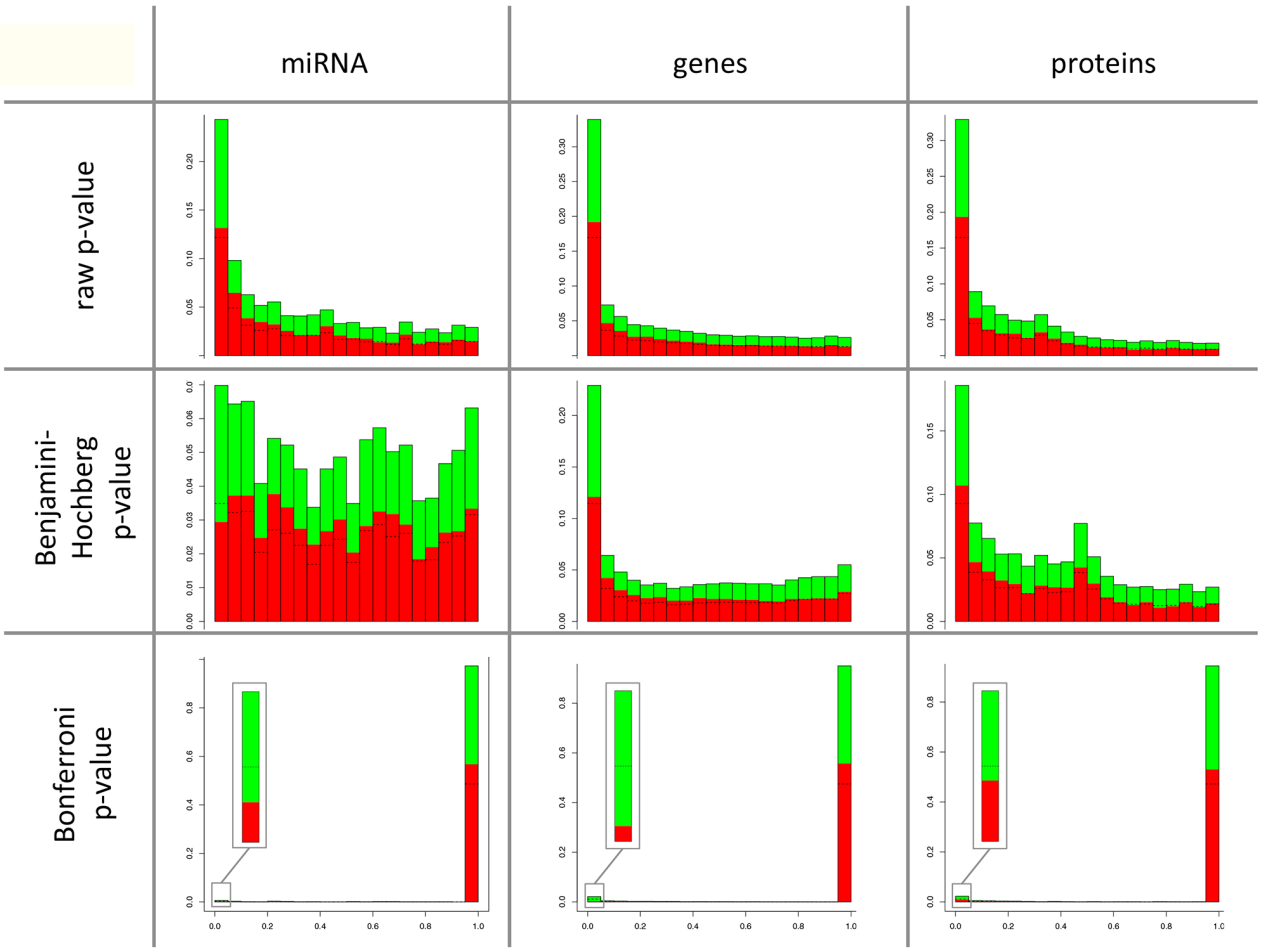
Comparison of the degree of deregulation between miRNAs, mRNAs and proteins The p-values are shown on the x-axis with each column combining five p-values and the frequency of the p-values are shown on the y-axis. The comparisons were done each by raw p-values, by p-values after Benjamini-Hochberg adjustment, and by p-values after Bonferroni adjustment. The direction of regulation is indicated in green for miRNAs, genes, and proteins with a decreased level in cancer and in red for increased level in cancer as compared to control lung tissue. There is a comparable distribution of raw p-values for proteins, mRNAs or miRNAs. Benjamini-Hochberg adjusted p-values showed a distribution of miRNAs different from the distribution of mRNAs and of proteins. The number of proteins, mRNAs or miRNAs that showed an increased level in cancer was similar to proteins, mRNAs or miRNAs with a decreased level in cancer for each the proteomic, transcriptomic and miRNomic data. The more stringed Bonferroni p-values show a reduction of significant mRNAs, miRNAs and proteins with an increased level and more significant proteins, mRNAs or miRNAs with a decreased level in cancer.

Comparing the distribution of raw p-values of the paired t-test (comparing cancer to control) we found similar histogram shapes for proteomic, transcriptomic and miRNomic data (Figure 1 – first row). In contrast, Benjamini-Hochberg adjusted p-values showed a different distribution of miRNAs compared to mRNAs and proteins (Figure 1 – second row). For the more stringent Bonferroni adjusted p-values (Figure 1 – third row) we observed again a similar distribution of p-values for miRNAs, mRNAs and proteins and also the expected reduction of significant mRNAs, miRNAs and proteins. Only 15 of 2,549 miRNAs (0.6%), 752 of 34,687 mRNAs (2.2%) and 141 of 6,183 proteins (2.3%) were significant after Bonferroni adjustment. Comparing the ratio of up- and down-regulated miRNAs, genes and proteins, the Bonferroni p-values indicated more features with a significantly decreased level in cancer as compared to normal controls: 11 of 15 miRNAs (73.3%), 675 of 752 genes (89.8%) and 84 of 141 proteins (59.6%) showed a reduced abundance in tumors as compared to controls.

**Figure 2:**
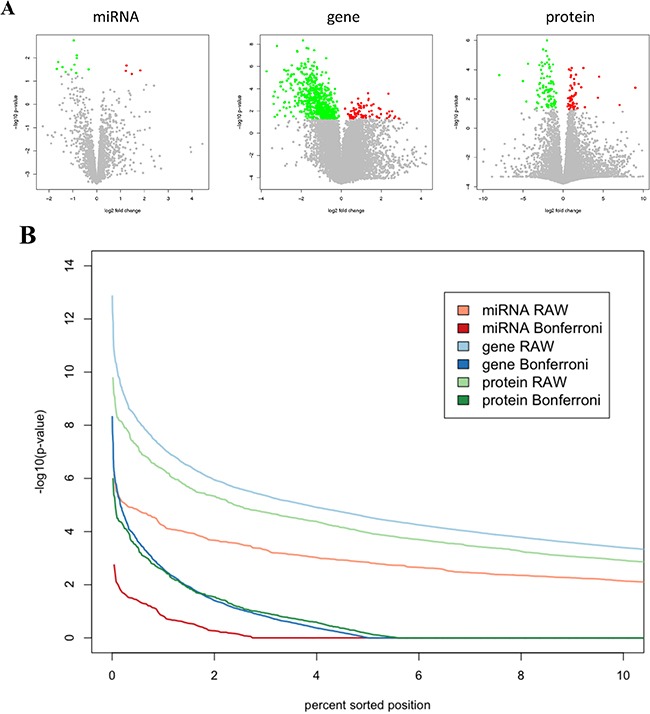
**A.** Volcano plots for miRNAs, mRNAs and proteins. The log_2_ fold change is shown on the x-axis and the negative decade logarithm of the p-value is shown on the y-axis. Each feature, i.e. a single mRNA, miRNA or protein, is represented by a single dot. Significant features following Bonferroni adjustment are significant features are highlighted in dark grey indicating a decreased level in cancer each compared to the corresponding normal lung tissue. **B.** Comparisons of the effect strengths between miRNAs, genes and proteins. The significance values of each the proteomic, transcriptomic and miRNomic data were sorted in decreasing order. The figure shows the 10% most significant features (proteins, mRNAs and miRNAs). Without adjustment mRNAs are most significantly altered, followed by proteins and miRNAs. With Bonferroni adjustment, the 10% most significant mRNAs and protein show comparable p-values while the 10% most significant miRNAs have p-values indicting decreased significance.

Volcano plots in Figure 2A graphically demonstrate the unequal split in up- and down-regulation for proteins, mRNAs and miRNAs. These plots also allow for estimating different effect sizes of single omics features. In case of paired t-tests the effect size equals the value of the t-statistics divided by the square root of the cohort size, which is constant in our case. Lowest significance value of a miRNA that showed differential abundance between tumor and normal tissue was 0.0018. For an mRNA, lowest p-value was 4.7×10^−9^ and for a protein 10^−6^. Direct comparisons of unadjusted p-values reveal that significance values of proteins and of mRNAs differ by less than one order of magnitude while mRNAs and miRNAs differ by approximately three orders of magnitude (Figure 2B). Since we measured 2.4 fold more proteins than miRNAs and 5.6 fold more mRNAs than proteins – it is reasonable to compare the Bonferroni adjusted p-values. As demonstrated in Figure 2B the significance of miRNAs that are differentially expressed between tumor and control tissue was still substantially below the significance of mRNAs or proteins. Adjusted significance values of mRNAs and proteins matched well.

### Multivariate analysis of single omics entities

**Figure 3:**
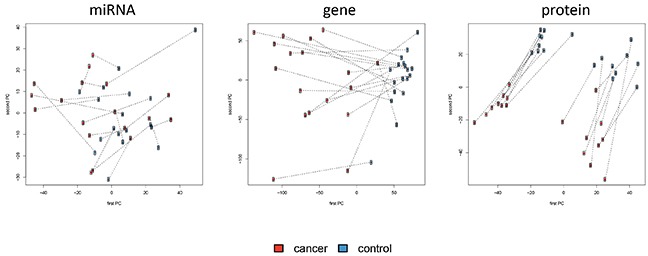
Principal Component Analysis of miRNA, mRNAs and protein abundances with the first versus the second principal component shown Lung cancer samples are indicated by red dots and control samples by blue dots. Pairs of cancer and control tissue that stem from the same patients are indicated by dotted lines. Proteins show a clear separation not only between tumor tissue and normal lung tissue, but also between adenocarcinoma and squamous cell carcinoma tissues.

Different approaches enable the analysis and visualization of complex high-dimensional omics data sets, including hierarchical clustering or principal component analysis (PCA). Both methods largely confirmed the results of the single feature analyses. While miRNAs did not enable clustering or differentiation in cases and controls, proteins allowed nearly perfect clustering and genes actually separated the tumor tissues perfectly from the unaffected controls (Supplementary Figure S1). As result of the PCA we present 2-dimensional scatter plots of the first and second PC (Figure 3). Again, miRNAs do not reveal a clear pattern. In case of mRNAs, already the first PC allowed for almost perfect separation of cancer tissues from controls. With respect to proteins, the second PC perfectly segregated between the tumor tissue and the according normal control tissue.

### Genomic proximity of miRNAs, genes, and proteins

**Figure 4:**
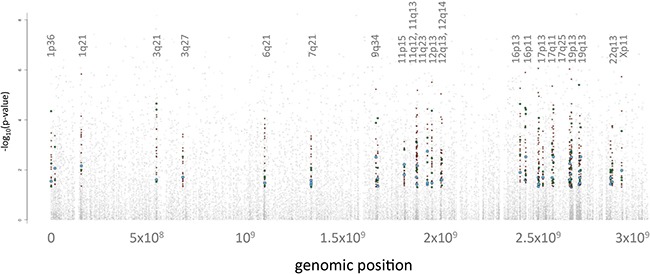
Integrated Manhattan Plot The genomic localization of features is shown on the x-axis, starting from chromosome 1 on the left hand side in increasing order to chromosomes 22, X and Y on the right hand side. The y-axis denotes the negative decade logarithm of the adjusted p-values. The 23 significant genomic regions each with at least one significant miRNA, mRNA and protein are shown. The miRNAs are indicated by blue dots, the mRNAs by red dots and the proteins by green dots. Since less miRNAs as compared to proteins and genes were included, the according dot sizes were increased to facilitate the interpretation.

A straightforward way of relating the obtained omics data to each other is to consider genomic proximity similar to Manhattan plots in genome wide association (GWA) studies. In analogy to Manhattan plots we extracted the genomic coordinates of the miRNAs, genes and proteins from public databases. We then projected these miRNAs, genes and proteins onto their genomic positions. Scanning the genome for clusters of significant events (i.e. features that showed a significantly different abundance between tumor and control lung tissues), we obtained 23 genomic regions enriched by deregulated features that contain at least one significant miRNA, gene and protein. Figure 4 provides an overview of the mapping results of the significant omics features, i.e. significant miRNAs, genes or proteins. We found an enrichment of significant features on chromosomes 11, 12, 16, 17 and 19 with chromosome 19p13 containing 86 significant features and 19q13 containing 49 significant features. In total, 21% of all single-omics discovered in this analysis can be attributed to chromosomes 19q13 and 19p13 (Supplementary Table S4).

### Signaling cascades - miRNAs and genes

**Figure 5:**
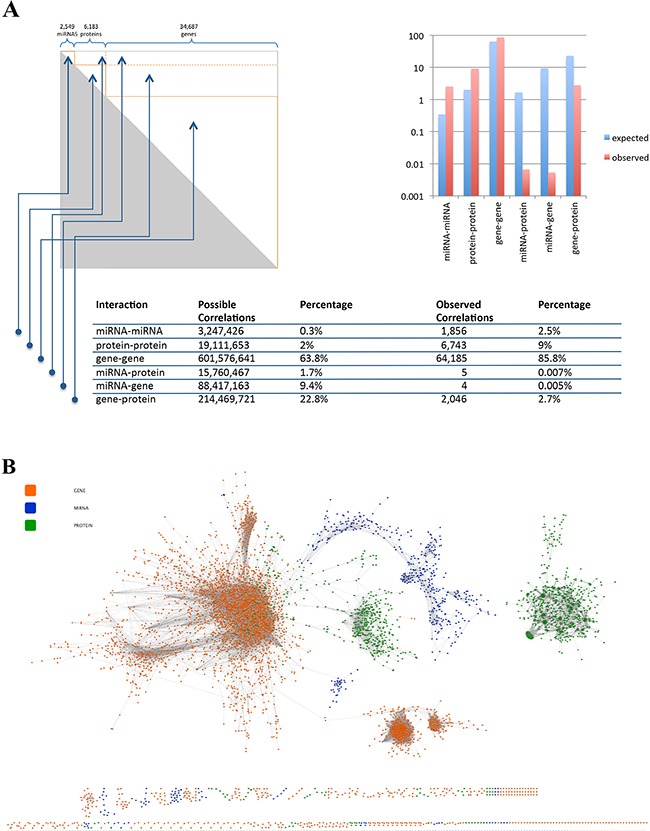
**A.** Pair-wise correlation matrix for all features with percentage wise distribution of intra- and inter-omics correlations. **B.** Ab-initio correlation network for all genes (green nodes), proteins (orange nodes) and miRNAs (blue nodes). Edges represent significant correlations following Bonferroni adjustment.

Since the lung cancer data set contains 43,419 features including 6,183 proteins, 34,687 genes and 2,549 miRNAs, there are 942 billion possible pair-wise interactions. Hence, Bonferroni p-value adjustment for a hypothesis test of all pair-wise interactions decreases the adjusted alpha level (from 0.05) to 5×10^−11^. Despite this low significance threshold we calculated 74,839 significant correlations among all pair-wise interactions (Figure 5A, Supplementary Table S5). Figure 5A shows the expected and the observed number of correlations between the different omics types. The largest difference between the observed and the expected frequency was found for the interactions between miRNAs and mRNAs. In detail, we found only five cases of miRNAs correlating significantly with mRNAs and 4 cases of miRNAs correlating significantly with proteins. The overall correlation network with 6,839 nodes and 74,839 edges is presented in Figure 5B. Since this figure does not allow for detailed inspection or even analysis of the network we provide a scalable version as pdf as well as the full network for investigation in Cytoscape for download at www.ccb.uni-saarland.de/supp_data/lung-multi-omics.

The low number of miRNAs significantly correlating with mRNAs and the overall large number of significant events, likely containing many false positives, prompted us to develop a network based approach including biological a-priori knowledge. Modeling the influence of miRNAs on mRNA, however, has to acknowledge the complexity of the interaction network between miRNAs and mRNAs with most miRNAs regulating larger sets of ‘competing’ genes and with many genes regulated by sets of miRNAs. The influence of a miRNA on one selected target mRNA depends on various factors including the abundances of all competing factors: a highly expressed miRNA that targets only this single gene has of course more influence than a less expressed miRNA that additionally regulates several other genes. We implemented a network approach to embed the mutual dependencies of mRNAs and miRNAs and the influence of the stoichiometry of all involved factors. A bipartite graph containing miRNAs and genes is generated with each edge connecting a miRNA (source node) with the targeted mRNA (target node). From a set of normal gene expression values the mutual influence of miRNAs and mRNA is calculated as edge weights. Given the interaction network and the edge weights calculated from the expression values of the control samples, we predict the influence of the miRNA expression changes between cancer and control onto the mRNA abundance changes. Then, the direction of the predicted changes is compared with the measured ones. Details of the algorithm are described in the Methods section.

In a first analysis, we considered all 37,443 miRNA-gene interactions from miRTarBase [4]. Training the model on control miRNA and mRNA we predicted the deregulation of significantly changed lung cancer genes. The quality of the forecast mainly depends on the number of miRNAs targeting mRNAs. Starting with mRNAs targeted by a minimal number of miRNAs (e.g. one or two miRNAs) and continuously increasing this number, the accuracy in correct predictions steadily increases. In detail, the deregulation of genes targeted by a single miRNA was correctly predicted in 48.1% of cases. Correct predictions were obtained for 66.7% of the genes targeted by 10 miRNAs, for 87.5% of the genes targeted by 15 miRNAs, and for 100% of the genes target by at least 16 miRNAs.

Since the miRTarBase contains a larger number of miRNA-mRNA interactions with weak support, i.e. miRNA-target gene interactions that are not validated by functional assays, we applied our new model in a second experiment to a high confidence interaction network that contains the 2,784 miRNA-mRNA associations that have been validated by functional assays [4]. The deregulation of genes targeted by a single miRNA was correctly predicted in 58.2% of cases, i.e. 184 significantly deregulated lung cancer genes have been correctly predicted. Correct predictions were obtained for 61.4% of the genes targeted by ≥2 miRNAs, i.e. 88 significantly deregulated lung cancer genes. Likewise, correct predictions were obtained for 75% of the genes targeted by ≥3 miRNAs (48 genes), for 86.7% of the genes targeted by ≥4 miRNAs (30 genes), for 90.9% of the genes targeted by ≥5 miRNAs (22 genes), and for 92.3% of the genes targeted by ≥6 miRNAs (12 genes).

**Figure 6:**
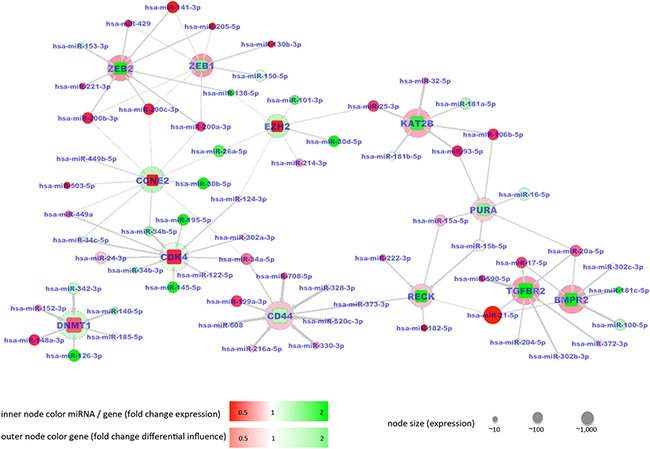
Network of 64 miRNAs indicated by circular nodes and 12 validated target mRNAs indicated by rectangles Spheres around the mRNA nodes indicate the fold change of mutual influence of miRNAs between normal lung and cancer samples. The mRNAs showed significantly altered abundances between lung cancer tissues and controls. Increased abundances are shown in red, reduced abundances in green. The shade of a color node color represents the fold change with a darker tone indicating a larger fold change between tumor and control tissue. Node sizes reflect the absolute expression of genes / miRNAs. Edges in the graph indicate validated targeting between miRNAs and mRNAs with the edge weight corresponding to the influence of a miRNA on a mRNA (*w* factors, see Materials). MiRNAs with increased color intensity, i.e. an increased fold change, frequently target several mRNAs. In all cases the color of a rectangle i.e. a validated target, is different from the color of the surrounded spheres indicating that increased mRNA abundance is associated with decreased miRNA abundance or vice versa.

In the latter case, the 12 correctly predicted genes have validated interactions with 64 miRNAs. The resulting interaction network with changes in mRNA abundance associated with changes of miRNA abundance contains 12 target genes including CD44, ZEB1, ZEB2, BMPR2, RECK, TGFBR2, PURA and KAT2B as correctly predicted down-regulated genes, and DNMT1, CCNE2, CDK4, and EZH2 as correctly predicted up-regulated genes in tumor tissue. A false prediction was only made for the up-regulated gene HIF1A, which is predicted as down regulated according to our model. The interaction network shown in Figure 6 demonstrates the different patterns of miRNA - mRNA relations: Down-regulation of CD44, ZEB1 or ZEB2 appears to be induced by several miRNAs, which show a low abundance. Up-regulation of CDK4 can be mainly attributed to miR-195-5p and miR-145-5p, both of which show a high abundance. All but one of the 64 miRNAs is belonging to pathways in cancer and negative regulation of apoptotic processes, as annotated in KEGG (Supplementary Table S6).

### Expression cascades – genes and proteins

Having modeled the influence of miRNAs on cancer genes, we addressed the relation between gene- and protein expression. The correlation between mRNA expression and abundance of the proteins encoded by the according genes was weak with a correlation coefficient of 0.11 (Supplementary Figure S2A). This result is consistent with previous observations that gene expression is far from being a perfect surrogate for protein expression [5]. Comparing mRNA and protein deregulation in cancer and control tissues by the AUC value, we found an increased correlation (0.64, p<10^−10^, Supplementary Figure S2B). In 80%, proteins and genes were both up- or down-regulated and in only 20% of cases up-regulated genes encoded down-regulated proteins or vice versa (Supplementary Table S7).

## DISCUSSION

We report a quantitative analysis of miRNA-, mRNA- and protein expression to further the discovery of altered signaling cascades. An important aspect of our study set-up is the autologous measurement, i.e. all omics data have been measured from the same tissue specimens. Also important for our study design is the paired analysis as reasonable means to increase the statistical power especially in due consideration of the large feature sets.

Many factors that influence the results of high-throughput studies can significantly impact the comparability between different omics studies [6]. These factors particularly include normalization approaches, hypothesis tests for discovering significantly altered features, and approaches for the p-value adjustment. Many of the features in our study were normally distributed (according to Shapiro-Wilk test), fulfilling the criteria for applying parametric t-tests. Notably, the t-test is not sensitive to deviations from the normality, especially when the deviations are moderate [7]. In addition, we controlled for the concordance of the t-test to the non-parametric Wilcoxon Mann-Whitney (WMW) test. In all cases we found significant correlations of t-test and WMW test with values of 0.92 for miRNAs, 0.93 for mRNA, and 0.85 for proteins. Analyzing the p-value adjustment, we observed substantial differences between the numbers of deregulated features and between the portions of up- and down regulations depending on the approach used for p-value adjustment. As shown in Figure 1 the fraction of significantly up- or down regulated cancer genes strongly differs between conservative Bonferroni adjustment and family-wise error rate based methods as Benjamini-Hochberg adjustment.

The analysis of single omics features indicates that miRNAs have a lower influence on the molecular pathogenic processes in lung cancer than mRNAs and proteins as shown by the effect size of miRNAs that was significantly lower than the effect size of mRNAs and proteins. Multivariate consideration by PCA and hierarchical clustering confirmed these results. To gauge the biological impact of miRNAs, mRNAs and proteins, it is, however, necessary to employ a combined analysis of the three omics data sets. To this end, we studied the genomic proximity of all deregulated features. A window-based approach identified 23 genomic regions with clusters of miRNAs, mRNAs, and proteins all of which were significantly altered in lung cancer as compared to controls. Copy number gains and losses in all identified regions have been previously described in either lung adenocarcinoma or squamous cell carcinoma [8–11]. For example, gains on 1q21 were found in more than 50% of adenocarcinomas and squamous cell carcinoma, and have been associated with a metastatic phenotype in the latter [8, 11]. For this region, we found 13 genes with increased expression, including cdc like kinase 2 (CLK2). As oncogenic kinase CLK2 is involved in regulating alternative splicing and has been found overexpressed in breast cancer and glioblastoma [12, 13]. Loss of 19p and gain of 19q is one of the most frequently found genetic aberrations in lung cancer [14]. In total, 21 % of deregulated features in our data mapped to 19q13 and 19p13, suggesting a role for genes and miRNAs in these regions for lung tumor genesis. In addition, for example miR-106b-5p, miR-93-5p and 200c-3p that were among the most abundant miRNAs in lung cancer, are located on chromosome regions 12p13 and 7q21, both of which are reported to be involved in cancer [21–23]. The miRNAs themselves have also been associated with lung cancer [24, 25].

To understand the influence of miRNAs on target genes, network based algorithms have been developed including ab-initio based methods that rely on measures such as the pair-wise correlation coefficient [15]. In our analysis the respective approach identified 74,839 significant pair-wise correlations despite a low alpha level (i.e. a low probability of rejecting the null hypothesis although it is true) of 5×10^−11^. Among the pair-wise correlations there were only four miRNA-mRNA pairs, none of which has been annotated in the miRTarBase. Methods that include available a-priori information as for example predicted miRNA-mRNA interactions have been developed to address problems associated with the ab-initio network approaches [16]. Zhang et al. proposed the SNMNMF (Sparse Network-Regularized Multiple Non-Negative Matrix Factorization) method that incorporates miRNA and mRNA values [16]. In other approaches, the strength of miRNA–mRNA interactions has been modeled by edge weights. Li and co-workers tested four measures, including the Pearson Correlation Coefficient, for defining edge weights towards maximization of a synergy score [17]. We implemented a linear model that incorporates stoichiometric effects. Our algorithm that scores the influence of miRNAs on mRNA performed particularly well when applied to experimentally validated miRNA-mRNA interactions and for mRNAs that were targeted by several miRNAs. For a scenario of at least 5 miRNAs, a significant change of mRNA abundance as result of altered miRNA patterns was correctly predicted in 92.3% of cases, only the prediction of a downregulated HIF1A was false and not supported by our data, that showed upregulation of HIF1A. Expression of HIF1A is tightly regulated on transcription and translation level, with increased levels of HIF1A in tumors including lung cancers and under hypoxic conditions [18, 19]. There are three possible explanations for the falsely predicted downregulation of HIF1A in the miRNA/mRNA network. First, the model leading to this prediction is based on experimentally validated target gene-miRNA interactions as reported in miRTarBase, so that known HIF1A regulating miRNAs are apparently overexpressed in lung cancer tissue, suggesting subsequent downregulation of HIF1A, which is not the case. Even if these miRNAs are upregulated in lung cancer tissue, it might be possible, that they are regulating other genes apart from HIF1A, which are not yet validated target genes and therefore not listed in the miRTarBase and not part of the prediction model. These assumed potential targets might minimize the effect of the upregulated miRNAs on the HIF1A mRNA due to simple stoichiometric reasons. Second, apart from the miRNA binding sites, there are several potential binding sites in the 3′UTR of HIF1A for proteins regulating HIF1A protein translation or mRNA half-life in both directions [19]. Therefore, a scenario where mRNA stabilizing proteins might be bound to the HIF1A mRNA, masking the miRNA bindings sites in a way, that miRISC complex cannot bind to the 3′UTR and therefore cannot exert its mRNA destabilizing function, is possible. In this case, even if HIF1A downregulating miRNAs are overexpressed, mRNA levels might not be affected. Third, other unknown regulatory mechanism can compensate the effect of miRNAs. In general, considering a scenario with mRNAs that are targeted by only one or two miRNAs, we obtained a decreased accuracy. This may be due to the following limitations of our approach: First, there are still miRNAs that have not been identified as targeting partner of a given mRNA or that have been not listed as such in the miRTarBase, which is the basis of our prediction approach. The higher the number of miRNAs that target a specific mRNA, the lower is the influence of each miRNA on the targeted mRNA. Second, our approach does not incorporate the influence of other regulatory factors that potentially act jointly with miRNAs such as transcription factors [20], methylation or other epigenetic alterations. Third, different miRNAs will likely have varying biological effects on different targets. Forth, the mutual interactions of miRNAs and mRNAs have of course a strong dynamic component. Adding the kinetics of miRNA-target gene interactions will further enhance the performance of the prediction algorithm. Respective approaches could potentially focus on feed-forward circuits, as proposed by Re and co-workers [20].

The network predicted by our approach consists of 8 downregulated and 4 upregulated genes, whose expression is potentially regulated by 64 interacting miRNAs. In general, downregulation of ZEB1, RECK, TGFBR2 and BMPR2, as well as upregulation of CDK4, CCNE2, EZH2 and DNMT1 has been shown in lung adenocarcinoma and/or squamous cell carcinoma [26–32]. Also deregulation of many of the miRNAs in our network is also known to contribute to NSCLC genesis, e.g. upregulation of miR-21 or downregulation of miR-145 [33]. One central part of our network is the association of downregulated ZEB1 and ZEB2 and the upregulated members of the miR-200 family, i.e. miR-200a/b/c, miR-141 and miR-429. The feedback loop of transcription factors ZEB1/2 repressing transcription of miR-200 family members, which in turn inhibit translation of ZEB1/2 on post-transcriptional level regulates epithelial to mesenchymal transition, a process important for cancer progression and metastasis [34]. In addition, through integration of known miRNA/target relationships from miRTarBase in our predicted NSCLC network, we uncovered novel interactions potentially relevant for lung cancer development. For example, upregulation of miR-21 and miR-92b and downregulation of their direct target RECK, an inhibitor of matrix metalloproteases, has been demonstrated in lung cancer and promotes cellular motility and proliferation [35]. In our network, we found that miR-222 and miR-182 might also contribute to RECK downregulation. Translational repression of RECK by miR-222 has already been shown for gastric cancer, and by miR-182 for prostate, breast and bladder cancer [36–39], but so far not for NSCLC. Another example is the downregulation of KAT2B (or PCAF) through upregulation of the miR-106b~25 cluster, which has been proposed to play a role in multiple myeloma by partially inactivating p53 [40]. While downregulation of miR-145 and simultaneous upregulation of CDK4 has been shown for NSCLC [33], we found that also downregulation of miR-195 might play a similar role in CDK4 translational regulation, as is already known for bladder and liver cancer [41, 42].

Taken together our analyses enable a comprehensive understanding of the influences of miRNAs on mRNA and protein abundances. Notably, the combined analysis of individual omics data sets in the present study is concordant to results in various previously published lung cancer studies. Profiling miRNAs, gene expression and proteins in a paired manner and thereby minimizing inter-individual changes, we were able to reconstruct relevant cascades involved in molecular pathology of lung cancer and to provide a more systematic and complete understanding of crucial biological mechanisms related to lung cancer. Our results emphasize the importance of autologous multi-omics studies overcoming challenges of current study set-ups.

## MATERIALS AND METHODS

### Patient characteristics and sample handling

Tissue specimens were collected during lung cancer resection as described previously [3]. In brief, samples were transferred into RNAlater TissueProtect Tubes (Qiagen) and incubated over night (4°C). RNAlater was removed and samples stored at −80°C. We collected 18 matched pairs of case and control tissue from 9 squamous cell lung carcinoma and 9 adenocarcinoma patients. Clinical details of these patients are given in Table 1 of Tenzer et al. [3]. Tissue specimens were obtained with patient informed consent from SHG Clinic, Völklingen, Heart Center. The study was approved by the local ethics committee (Ärztekammer des Saarlandes, 01/08).

### Multi-omics data generation

Experimental details for acquisition of proteomic and transcriptomic data for the 18 matched tumor/control specimens have been described previously [3]. While gene expression data is the same in the previous and the current study (34,687 genes), we applied an optimized and more sensitive method for discovery of differentially expressed proteins in the current analysis, which lead to an increased number of detected proteins (now 6,183 proteins in comparison to 3,328 proteins in the previous analysis). In detail, we repeated the entire proteome analysis starting from the same source material, with the following minor modifications: a) Sample preparation was optimized, including additional washing steps resulting in cleaner samples [43]; b) NanoUPLC conditions were optimized by adding 3% DMSO to the mobile phase, which improves ionization efficiency by about 2-fold [44]; c) Drift-time specific collision energies were optimized to further improve fragmentation efficiency; d) For the analysis of the new sample set, the instrument was freshly serviced, including the installation of a new detector, resulting in approx. 2-fold increase in absolute sensitivity.

The combined effect of the above differences resulted in the observed increase in identified and quantified proteins. In addition to the improved protein analysis, we performed miRNA expression profiling of these tumor and control specimens to add an additional regulatory layer to our multi-omics data set. We screened the expression of 2,549 mature human miRNAs as annotated in the miRBase version 21 using the Agilent microarray system according to manufacturers instructions as described previously [26].

### Basic statistical analysis

Raw expression values of miRNAs, transcripts, and proteins were calculated according to manufacturers instruction. All further calculations have been performed using R (version 3.0.2). Before further processing, expression values were quantile normalized for each omics separately. To assess whether data are normally distributed Shapiro-Wilk tests were applied. To estimate the effects between cancer and control samples for each miRNA, gene and protein, t-test, Wilcoxon-Mann-Whitney test and the area under the receiver operator characteristic curve were computed. To account for different feature set sizes p-values were adjusted by Benjamini-Hochberg and Bonferroni adjustment. Principal Component Analysis (PCA) was carried out using the prcomp package and hierarchical clustering was done using the heatmap.2 function.

### Analysis of genomic proximity

To discover genomic regions that are enriched for statistically significant single-omics features we first extracted the genomic coordinates of miRNAs, genes and proteins from public repositories [27]. The genomic positions have been mapped to consecutive positions between 1 and 3×10^9^, where chromosomes have been ordered from 1 to 22, X and Y. Then a window of 1 million bases was shifted over the 3 billion bases and for each window position the number of significant miRNAs, genes and proteins was calculated. Only features with adjusted p-value below 0.05 were considered to be significant. We reported regions with at least one significant miRNA, gene and protein and with the additional side condition that more than 10 significant features had to be located inside the region. Results are graphically represented as Manhattan plots where the X-axis denotes the genomic position and the Y-Axis the negative decadic logarithm of the p-value.

### Modeling the joint influence of miRNAs on target genes

Core of the mutual analysis of genes and miRNAs is a novel network algorithm that incorporates the stoichiometry of miRNAs and target genes. The network consists of miRNAs and genes as nodes and miRNA and gene nodes are connected by an edge if the gene is a target of the respective miRNA. For this network the algorithm estimates the influence of each miRNA on each target gene. Given a set of genes, miRNAs and targets, the algorithm works as followings:

For each miRNA *m*_*i*_, all target genes *g*_*j*_ are considered. The influence of each miRNA *i* on each targeted gene *j* is computed as

$$w_{\text{ij}}=\frac{e(g_{j})}{\sum_{k=1}^{l}e(g_{k})},\text{where}$$

*e*(*g*_*j*_) represents the expression of the targeted gene *j*, *l* is the number of target genes of *m*_*i*_ and hence the sum in the denominator runs across all target genes of this miRNA. The above quotient is used as edge weight for edges connecting *m*_*i*_ and *g*_*j*_.

Accordingly, the joint influence of all miRNAs on one gene *j* can roughly be approximated by the following linear function :

$$s(g_{j})=\sum_{k=1}^{z}w_{\text{kj}}e(m_{k})$$

This joint influence score summarizes the influence of all miRNAs that are targeting gene *j*. Here, the influence of each miRNA is approximated by the product of its weight and its abundance given by the corresponding expression value.

This procedure takes the complex interaction network between miRNAs and genes and their expression (miRNA and mRNA) into account. The novelty of this approach is the fact that the presented weighting scheme incorporates the abundance of all competitive genes that are targeted by the same miRNA and that are competing for binding this miRNA. This kind of simplified stoichiometric approach hence mirrors the mutual influence and interference of genes and miRNAs. Thus, the model also allows to distinguish between miRNAs that have a big influence on a gene such as a highly expressed miRNA that only targets one lowly expressed gene and miRNAs that have substantially lower influence on gene expression such as a low-abundant miRNA that is targeting several highly expressed genes.

We inferred the edge weights from the gene expression of control samples. Based on these edge weights we computed the joint influence score *s*^*N*^(*g*_*j*_) of miRNAs on all target genes *g*_*j*_ in control samples. Next, we used the statistical model to calculate the joint influence score *s*^*C*^(*g*_*j*_) of miRNAs in cancer samples. Genes with an increased joint influence score $\left(s^{C}(g_{j})-s^{N}(g_{j})\right)>0$ in cancer compared to controls are expected to have lower expression in cancer, and vice versa, genes with decreased joint influence score $\left(s^{C}\left(g_{j}\right)-s^{N}\left(g_{j}\right)\right)<0$ should have higher expression.

As resource for miRNA-target gene interactions we used information extracted from miRTarBase version 6 [4]. Both, all targets from miRTarBase and only the strong functionally validated interactions were considered separately and the performance of both miRNA target sets was compared.

### Determining the influence of genes on proteins

To determine whether gene expression matches abundance of proteins we mapped the swissprot identifiers to gene names. Next, we extracted all significantly changed genes and proteins. First, we compared the median expression across all samples and separately for cancer and control samples between matched pairs of proteins and genes. Since the absolute values did not correlate, we compared the difference in gene and protein expression between cancer and control samples. We compared all 1,122 gene-protein pairs where both, genes and proteins coded by these genes had raw t-test p-values below 0.05. For these, we correlated the differential expression (AUC values) of genes to the differential expression of proteins.

## SUPPLEMENTARY FIGURES AND TABLES
















