# Supplementary material for: Paired proteomics, transcriptomics and miRNomics in non-small cell lung cancers: known and novel signaling cascades
Source: Oncotarget. 2016 Aug 31;7(44):71514–25. doi: 10.18632/oncotarget.11723 (PMC5342097; doi:10.18632/oncotarget.11723)
Supplement: Supplementary file 1 [file oncotarget-07-71514-s001.pdf]

Paired proteomics, transcriptomics and miRNomics in non-small cell lung cancers: known and novel signaling cascades

SUPPLEMENTARY FIGURES AND TABLES

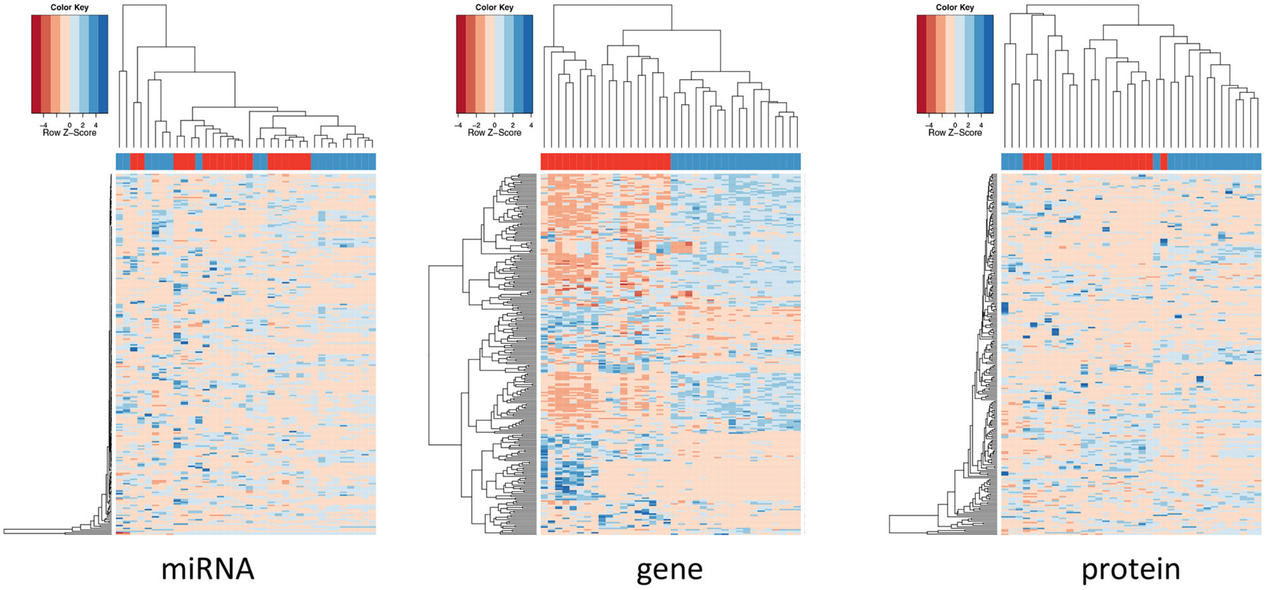

Supplementary Figure S1: Result of the hierarchical clustering with 100 most variable miRNAs / genes / proteins. High and low expression are represented in blue and red, respectively. Each feature was transformed to z-score scale. Blue and red between the heat map and the dendrogram on top indicate cancer and control samples, respectively.

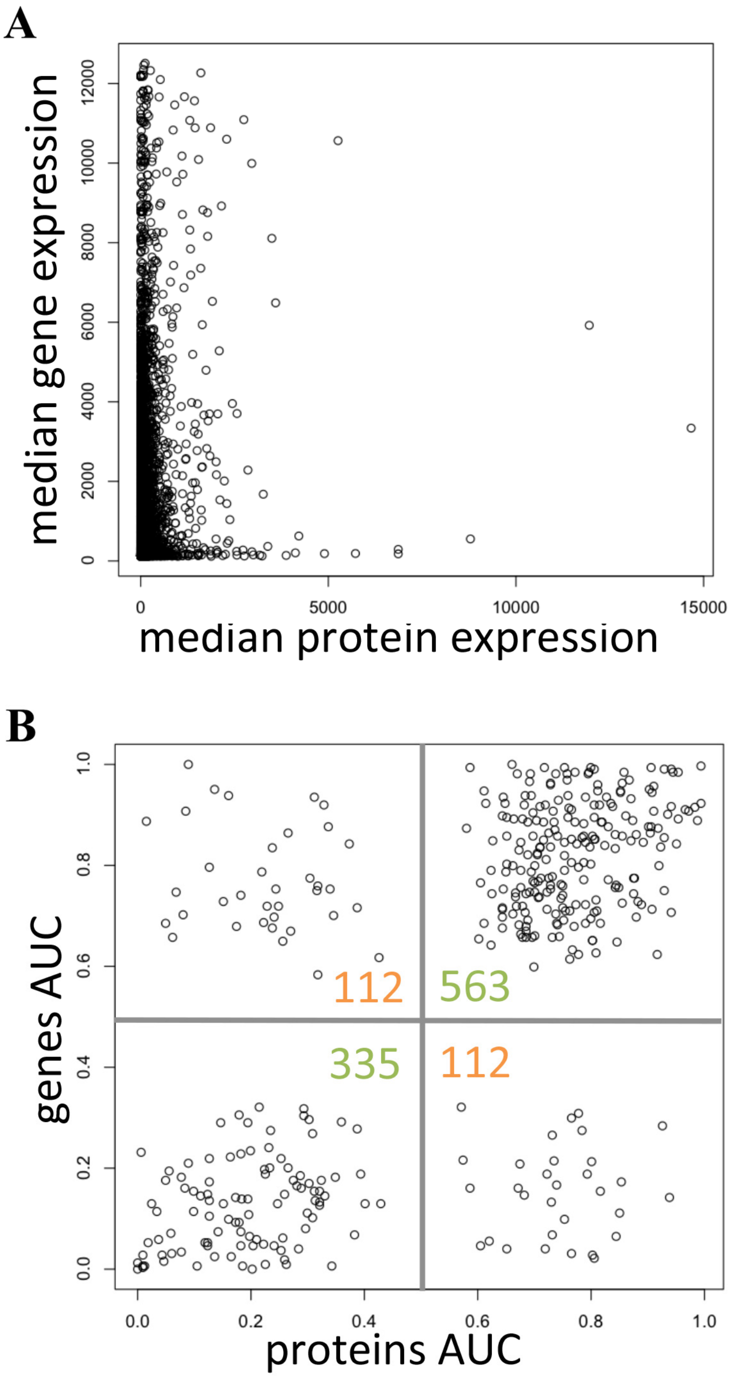

Supplementary Figure S2: Correlation of the absolute expression intensity of genes and matching proteins (panel A) and for deregulation of genes and proteins in cancer and controls (panel B). Numbers represent actual number of points in each sector.

Supplementary Table S1: Performance metrics for miRNAs.

See Supplementary File 1

Supplementary Table S2: Performance metrics for mRNAs.

See Supplementary File 2

Supplementary Table S3: Performance metrics for proteins.

See Supplementary File 3

Supplementary Table S4: Genomic hot-spots that are enriched for miRNAs, mRNAs and proteins.

See Supplementary File 4

Supplementary Table S5: All significant pair-wise interactions.

See Supplementary File 5

Supplementary Table S6: Enriched biochemical pathways and categories.

See Supplementary File 6

Supplementary Table S7: Comparison of gene to protein expression.

See Supplementary File 7
